# The Impact of Diclofenac Suppositories on Post-Cesarean Section Pain: A Systematic Literature Review

**DOI:** 10.1155/anrp/5457722

**Published:** 2025-03-16

**Authors:** Sara Agyemang Antwi, Prince Kwabena Agyemang Antwi, Samuel Akwasi Adarkwa, Kwesi Boadu Mensah, Eric Woode

**Affiliations:** ^1^Department of Pharmaceutical Sciences, Kumasi Technical University, Kumasi, Ghana; ^2^African Institute of Business and Leadership Excellence, Kumasi, Ghana; ^3^Department of Statistical Sciences, Kumasi Technical University, Kumasi, Ghana; ^4^Department of Pharmacology, Kwame Nkrumah University of Science and Technology, Kumasi, Ghana; ^5^Department of Pharmacology, University of Health and Allied Sciences, Ho, Ghana

**Keywords:** analgesia, Cesarean section, diclofenac, pain measurement, visual analogue scale

## Abstract

**Background:** Managing postoperative pain after Cesarean section is imperative, as acute postoperative pain is considered a risk factor for chronic postoperative pain. We investigated the role of diclofenac suppositories in postoperative pain management after Cesarean section.

**Methods:** For this systematic review, we searched PubMed, Scopus, the Cochrane Library, Google Scholar, and two other clinical trial registers from database inception up to July 23 to July 26, 2024. We included randomized controlled trials and other studies in which diclofenac suppositories were administered as an intentional intervention. We excluded studies not reported in English and without a focus on the principal medicine (diclofenac suppository). Two researchers independently chose studies and assessed the risk of bias using RoB-2, following the PRISMA-2020 guidelines. Primary outcomes included pain severity or intensity measured with validated clinical scales. We synthesized the studies narratively. The PICO was used to generate the research question: Population—Cesarean section patients, Intervention—diclofenac suppository, Comparison—opioids, Outcome—lower pain scores and a reduced need for more pain medications, Research question—the effectiveness of diclofenac suppositories in preventing postoperative pain and reducing the consumption of pain medicines in Cesarean section patients.

**Findings:** From an initial pool of 203 records, 20 records were selected for review. Notably, discrepancies in the study design and reporting were observed. This raised concerns about the consistency and reliability of the results obtained from the different studies. The visual analogue scale (VAS) emerged as the frequently used pain assessment tool. Diclofenac suppository was compared against other treatments under three categories: placebo, other nonsteroidal anti-inflammatory drugs (NSAIDs), and opioids or opioid-like medicines. The findings revealed that diclofenac suppository was effective in reducing pain compared to placebo and hence, minimized the need for opioids. The concept of combining pain medicines for postoperative management, known as multimodal analgesia, was central to most of the studies.

**Interpretation:** Combination of diclofenac suppositories with other pain relief medicines reduces the need for rescue pain medicines, which are usually opioids such as morphine, meperidine, or pentazocine.

**Clinical Implications:** Patient satisfaction can be improved with these enhanced pain management strategies. Also, reliance on opioids for postoperative pain management and its related side effects will be reduced. This research reinforces the importance of multimodal analgesia in postoperative pain management. The findings also open pathways for further clinical trials to explore the appropriate combinations, dosages, and administration of NSAIDs for specific surgical populations and settings. Future research should focus on standardizing methodologies and addressing risk of bias to enhance reliability of findings related to diclofenac suppository and multimodal analgesia.

## 1. Introduction

### 1.1. Rationale

Managing postoperative pain after Cesarean section is imperative, as acute postoperative pain is considered a risk factor for chronic postoperative pain [1, 2] and psychosocial disorders in mothers. It affects the healing time and may also have an impact on breastfeeding. The American Pain Society and the American Society of Anesthesiologist (ASA) recommends the use of opioids postoperatively. Adjunctive treatment, including nonsteroidal anti-inflammatory drugs (NSAIDs) and/or acetaminophen, may be administered preoperatively for Cesarean section patients as systemic pharmacologic therapy. This is accepted in addition to transversus abdominal plane block (regional anesthetic technique) and epidural with a local anesthetic (with or without opioid) or intrathecal opioid as neuraxial anesthetic techniques, local techniques involve local anesthetic at incision [3].

Currently, there is no best choice for practice regarding the time to administer diclofenac suppositories, whether preoperatively or postoperatively. It is unclear what the general practice is and whether preoperative administration is associated with better pain relief postoperatively and greater patient satisfaction than immediate postoperative administration or administration upon request. Gathering reliable data in a transparent and concise way is essential for empowering women to make informed decisions and enhancing practice guidelines. This approach facilitates objective counseling for women scheduled for Cesarean sections. As part of this review, a synthesis of all available randomized controlled trials (RCTs) and related studies is conducted to evaluate the effectiveness of diclofenac suppositories in managing postoperative pain.

The findings of this review will help optimize the perioperative use of diclofenac suppositories, leading to a decrease in the occurrence of severe acute postoperative pain. No systematic review has assessed the role of the suppository diclofenac in reducing acute postoperative pain or its impact on the overall opioid consumption of patients after Cesarean section.

### 1.2. Objectives

The primary objective of this review was to examine the role that diclofenac suppositories play in postoperative pain management after the Cesarean section. Thus, this systematic review aimed to answer the following questions.

  What is the impact of diclofenac suppositories on postoperative pain?
  What is the impact of diclofenac suppositories on opioid consumption?
  What is the appropriate strategy for the use of diclofenac suppositories to combat postoperative pain?
  Does diclofenac suppository provide satisfactory pain relief in comparison to other nonopioids?
  Do diclofenac suppositories provide satisfactory pain relief equal to opioids?

## 2. Methods

### 2.1. Eligibility Criteria

#### 2.1.1. Study Types

We included all published RCTs and other study types, such as cross-sectional and prospective cohort studies, that addressed the research question. The inclusion of all published RCTs as well as other research types including cross-sectional and prospective cohort studies increased the comprehensiveness of the review. RCTs have the capacity to reduce bias and demonstrate causal linkages through randomization [4]. They are regarded as the gold standard in clinical research. However, other study types might also offer insightful information.

#### 2.1.2. Language Selection

The search was conducted in English. This is because English language is the universally accepted language for majority of publications.

#### 2.1.3. Time Range

No studies were excluded based on the date of publication to ensure that both older and newer research which hold significance is not missed.

#### 2.1.4. Dosage Forms

We excluded studies that used diclofenac in any other dosage form aside from suppositories. This was to help maintain a specific formulation of interest at a time. Different dosage forms could have different pharmacokinetic and pharmacodynamic profiles which would have been difficult to reconcile if all dosage forms were included. We also excluded studies in which diclofenac suppository was merely a baseline adjunct treatment but not the principal medicine of interest. This approach was crucial for drawing valid conclusions about the efficacy of diclofenac suppositories.

#### 2.1.5. Participant Types

We included women of any age who had either an emergency or elective Cesarean section performed under regional or general anesthesia, classified as ASA I or II. Studies that used an accepted pain assessment tool according to the American Pain Society guidelines.

#### 2.1.6. Types of Interventions

Despite the ASAs' recommendation to give opioids orally rather than intravenously (IV) for postoperative pain relief in individuals who are able to do so [3], most patients will find it difficult to take orals especially due to nausea and vomiting [5]; which has led to a massive use of injectable analgesics and suppositories postoperative. However, because of the society's stance on injectables, suppositories became the focus for this study.

Studies in which interventions described the use of diclofenac suppositories either as a one-time therapy or at a particular frequency. The interventions may have been monotherapy or in combination with other analgesics.

The main categories of approaches were diclofenac suppository single-dose preoperative; diclofenac suppository single-dose immediate postoperative; diclofenac suppository 4-hourly to 12-hourly; and diclofenac suppository therapy compared with intrathecal morphine, IV acetaminophen (paracetamol), acetaminophen suppository, tramadol suppository, morphine suppository, IV/IM meperidine (pethidine), and IM pentazocine.

#### 2.1.7. Types of Outcome Measures

Primary outcomes included the following:

Pain scores are measured with a numerical pain rating scale, visual analog scale (VAS), verbal rating scale, or a combination of these.

Secondary outcomes: Patient satisfaction, side or adverse effects and request for rescue analgesic medicines.

In summary, these criteria were adopted to enhance the reliability of the findings with a specific focus on diclofenac suppositories. This will also ensure that conclusions drawn from the review are relevant to clinical practice.

### 2.2. Information Sources

We conducted electronic searches for eligible studies within each of the following databases and registries:

Scopus (entries from 1994 to 2024) accessed on 24^th^ July 2024.

PubMed (entries from 1995 to 2023) accessed on 24^th^ July 2024.

Cochrane Central Register of Controlled Trials (entries from 1994 to 2024) accessed on 25^th^ July 2024.

The US National Institutes of Health Ongoing Trials Register ClinicalTrials.gov (https://www.clinicaltrials.gov/) was accessed on 25^th^ July 2024.

The International Clinical Trials Registry Platform (https://apps.who.int/trialsearch/) 2013 to 2015 was accessed on 26^th^ July 2024.

The European Union Clinical Trials Register (https://www.clinicaltrialsregister.eu/) was accessed on 26^th^ July 2024.

Google Scholar was accessed on 26^th^ July 2024.

### 2.3. Search Strategy

#### 2.3.1. Database Selection

##### 2.3.1.1. Primary Database 1: Scopus

  Rationale: Scopus provides comprehensive coverage of medical literature, including international journals and ability to handle complex Boolean searches.
  Time Range: 1994–2024 (30 years).
  Rationale: This time range captures evolution of practice while maintaining relevance to current clinical standards. The 30-year window aligns with major developments in postoperative pain management protocols. The time range was obtained based on the literature that was finally selected for the systematic literature review.

###### 2.3.1.1.1. Search Strategy

  Query 1: Suppository Administration
  (suppository OR supp⁣^∗^) AND (diclofenac) AND (postoperative OR postoperative OR post operative) AND (caesarean⁣^∗^ OR Cesarean⁣^∗^ OR “c section” OR “c-section”) AND (pain OR analgesi⁣^∗^)
  Query 2: Rectal Administration
  (rectal OR rectally) AND (diclofenac) AND (postoperative OR postoperative OR post operative) AND (caesarean⁣^∗^ OR Cesarean⁣^∗^ OR “c section” OR “c-section”) AND (pain OR analgesi⁣^∗^)

###### 2.3.1.1.2. Limitations

Language Restriction (English language only) was to ensure that accurate interpretation without translation bias would be obtained. Moreover, English language is the predominant language of international medical literature. This approach also reduced the resource requirements for translation.

Considering document types, we decided to use Journal articles only and excluded conference abstracts, letters, editorials. This was to enable us focus on peer-reviewed complete research. Also, this choice ensured access to full methodological details and facilitated quality assessment. Only final publications were chosen. This ensured that articles that were included had received a complete peer review. This approach also helped us to avoid duplicate inclusion of preprints and maintain quality control.

The field restrictions were abstract, title, and keywords. These restrictions increased the precision by focusing on relevant content, reduced noise from incidental mentions in full text and maintained balance between sensitivity and specificity.

Search period and results: The search was performed on 24^th^ July 2024. Twenty-two (22) results were exported.

##### 2.3.1.2. Primary Database 2: PubMed

PubMed was selected as a database because of direct access to MEDLINE's biomedical literature, comprehensive coverage of clinical research, integration with Medical Subject Headings (MeSH) terminology, free accessibility enhancing reproducibility, and strong coverage of clinical trials and interventional studies.

  Time Range: 1995–2023 (28 years)

###### 2.3.1.2.1. Search Strategy

  Query 1: Suppository Administration
  ((diclofenac) OR (diclofenac[Title/Abstract])) AND ((suppository) OR (suppository[Title/Abstract])) AND ((postoperative pain) OR (postoperative[Title/Abstract] AND pain[Title/Abstract])) AND ((Cesarean section) OR (caesarean[Title/Abstract]) OR (Cesarean[Title/Abstract]))
  Query 2: Rectal Administration
  ((diclofenac) OR (diclofenac[Title/Abstract])) AND ((rectal) OR (rectal[Title/Abstract])) AND ((postoperative pain) OR (postoperative[Title/Abstract] AND pain[Title/Abstract])) AND ((Cesarean section) OR (caesarean[Title/Abstract]) OR (Cesarean[Title/Abstract]))

###### 2.3.1.2.2. Limitations

Abstract Availability was a filter that was applied. This ensured sufficient information for initial screening, facilitated assessment of study relevance, improved efficiency of review process, and reduced time spent retrieving potentially irrelevant full texts.

English language filter was applied as was done for the Scopus database. This approach also aligns with international publication standards.

Search period: This search was performed on 24^th^ July 2024. Fifteen (15) results were exported.

##### 2.3.1.3. Primary Database 3: Cochrane Library

Cochrane Library is the gold standard for systematic reviews and meta-analyses, specialized in controlled clinical trials. This database has a high methodological rigor in indexing and a comprehensive coverage of healthcare interventions. It also has built-in quality assessment tools.

###### 2.3.1.3.1. Cochrane Search Strategy

  Time range: 1994–2024 (30 years).
  Query 1: Suppository Administration.
  MeSH descriptor: [Diclofenac].
  MeSH descriptor: [Suppositories].
  MeSH descriptor: [Pain, Postoperative].
  MeSH descriptor: [Cesarean Section].
  (caesarean OR Cesarean).
  Query 2: Rectal Administration.
  MeSH descriptor: [Diclofenac].
  MeSH descriptor: [Administration, Rectal].
  MeSH descriptor: [Pain, Postoperative].
  MeSH descriptor: [Cesarean Section].
  (caesarean OR Cesarean).
  Search period and results - 25^th^ July 2024.
  Query 1: Forty-one (41) reports exported.
  Query 2: Four (4) reports exported.

##### 2.3.1.4. Primary Database 4: ClinicalTrials.gov.

This website captures ongoing and recently completed trials, identifies potential publication bias, provides insight into current research trends. It is mandatory registration for US trials but also has international trial coverage.

  ClinicalTrials.gov Search Strategy.
  Structured Search Parameters.
  Condition/Disease: Cesarean section.
  Intervention: Diclofenac suppository.
  Other Terms: Caesarean section; pain.
  Search period: The date on which this search was performed was July 25, 2024. Two (2) results were retrieved. One was already captured by the Scopus search, and the other was still enrolling participants.

##### 2.3.1.5. Primary Database 5: WHO ICTRP

This global trial registration platform aggregates data from multiple national registries, captures international research activity, and provides comprehensive trial monitoring. This site is essential for identifying geographical variations in research.

  Time Range: 2013–2015.
  WHO ICTRP (https://apps.who.int/trialsearch/) Search Strategy.
  Query 1: Suppository Administration.
  Primary Search Terms: diclofenac AND suppository AND postoperative AND pain AND Cesarean section.
  Query 2: Rectal Administration.
  Primary search terms: Rectal AND diclofenac AND postoperative AND pain AND Cesarean section.
  Search Period (ICTRP) and results—search was done on July 26, 2024, and Four (4) trials were exported.

##### 2.3.1.6. Primary Database 6: EU Clinical Trials Register

This register has European-specific trial documentation, regulatory compliance tracking, and detailed protocol information. It is mandatory registration for EU trials.

  EU Clinical Trials Register Strategy (https://www.clinicaltrialsregister.eu/).
  Search terms: Full text search: diclofenac AND (suppository OR rectal) AND Cesarean.
  Trial Status: All.
  Trial Phase: All.
  Search period and results—search was done on 26^th^ July 2024 and no relevant trials identified.

##### 2.3.1.7. Primary database 7: Google Scholar

  Google scholar captures grey literature, finds cited references, and enables forward citation tracking.
  Google Scholar Search Strategy.
  Primary Terms: diclofenac suppository postoperative pain Cesarean section rectal diclofenac postoperative pain Cesarean section.
  Custom range: All years.
  Sort by: Relevance.
  Search period—26^th^ July 2024 and thirty-one (31) results were exported.

### 2.4. Selection Process

Two researchers (SAA and PKAA) independently screened the titles and authors of all the records retrieved from the databases and registries. Duplicate records were eliminated through the first screening procedure, as were out-of-scope records and records that were simply unsuitable for the review. The resulting records after the first screening were subsequently screened according to the inclusion criteria, and the titles and abstracts were used for inclusion in the review. Inconsistencies were discussed until a consensus was obtained. Articles were flagged as yes, no or maybe. The term “maybe” was assigned to articles that did not fully meet the inclusion criteria. All articles that were assigned a “Yes” were subsequently included, and a review of their full text was performed. If necessary, a third researcher was consulted to make the final decision. Next, the researcher (SAA) independently screened the full-text articles for synthesis. PKAA reviewed the results of the full-text synthesis.

### 2.5. Data Collection Process

After modifying the data collection forms used by Yichun Gu and colleagues [6] and Michelle Palazzuolo and colleagues [7], we created a data extraction form specifically for this systematic review. One author used the form to extract data from eligible studies, while another author reviewed the extracted data. Any discrepancies were addressed through discussion. If any information was unclear, we consulted a third reviewer.

### 2.6. Data Items

Primary outcomes were categorized as pain intensity or severity, pain during the immediate postoperative period, and subjective reporting of pain.

#### 2.6.1. Methods of Outcome Measurement and Assessment

Any of the measures and assessment tools accepted by ASA, which was further accepted by the American Pain Society as appropriate for pain measurement, were eligible for inclusion [3]. For these scales, pain was subclassified into no pain, 0, mild pain, 1–3, moderate pain, 4–6 and severe pain; 7–10 or a mean score was used for analysis.

#### 2.6.2. Timing of the Outcome Measurement

Studies that assessed pain intensity or severity within 24–48 h after surgery were eligible. According to other studies, this is a critical period for optimizing pain management because the anesthesia would have worn out. There were no limitations on the frequency of pain assessments. We collected information on the characteristics of the included studies, and the results were structured as follows.

##### 2.6.2.1. Study Design and Characteristics

- Study context (location) and duration
- Study design (e.g., double-blind RCT, controlled trial, cross-sectional study, etc.)
- Funding sources, if available

##### 2.6.2.2. Patient Group Characteristics

- Qualitative descriptions of each category used (e.g., first-time Cesarean section, elective or emergency Cesarean section patients, age, etc.)
- The control group category used in the analysis and the specific characteristics that defined them as the control group
- Methods of data collection (e.g., retrospective methods, recall; time points at which exposure data were collected)
- Sample size for each exposure group at each measurement point included in the analysis
- Dose or strength of interventional drugs used and the route of administration
- Any additional parameters used to define each category or exposure measure
- Any further data that characterize and quantify the different patterns within the groups
- Duration or length of the exposure period at baseline and follow-up (directly reported or data that can be used for calculations)

### 2.7. Risk of Bias (RoB) Assessment

We assessed the RoB in the included studies using the revised Cochrane ‘RoB' tool for randomized trials (RoB 2.0) [8, 9]. RoB 2.0 is organized into a specific set of bias domains, each addressing different elements of trial design, execution, and reporting. Each domain includes a set of ‘signaling questions' designed to gather information about aspects of the trial that are pertinent to the RoB. Based on the responses to these questions, a judgment is made regarding the RoB for each domain. The judgment may indicate a ‘Low' risk, a ‘High' risk, or highlight ‘Some concerns' regarding bias. [8, 9]. RoB 2.0 addresses five key domains of bias: bias from the randomization process, bias due to deviations from intended interventions, bias from missing outcome data, bias in outcome measurement, and bias in the selection of reported results. Two review authors collaboratively applied the tool to each included study, documenting supporting information and justifications for their RoB judgments in each domain. Any disagreements in judgments or justifications were resolved through discussion to reach a consensus between the two authors. If consensus could not be achieved, a third author was consulted. In accordance with the RoB 2.0 guidelines, an overall ‘RoB' judgment (low risk, some concerns, or high risk) was determined for each specific outcome, based on the highest RoB observed in any of the assessed domains for each study [9].

### 2.8. Synthesis

Due to the heterogeneity of the studies included, the synthesis was performed narratively. Interventions were categorized into 4 dimensions:

Diclofenac suppository only vs control studies, combination therapy of nonopioids, diclofenac suppository vs opioids.

Meta-analyses were not possible due to the variability of the interventions (clinical diversity) [10, 11], settings, study designs and outcome measures. We examined the methods and results sections of the published papers by addressing the following questions:

  What things seem to be agreed upon by most of the papers?
  What are the areas of disagreement?
  What theories, hypotheses, or concepts seem to be coming up repeatedly?
  How are they connected or disconnected from each other?
  What types of research designs have been used?
  We also leveraged our knowledge of the clinical area to identify instances where trial investigators had omitted commonly used outcome measures.

Scopus and PubMed are the largest databases from which most studies can be identified. In addition, the Cochrane Library and the other aforementioned registries were searched for studies to include in this systematic literature review to reduce the risk of reporting biases [12].

We used Cochrane guidelines on systematic reviews and GRADEpro software to assess certainty in the body of evidence for the outcomes of all the included studies (Table 1).

**Figure 1:**
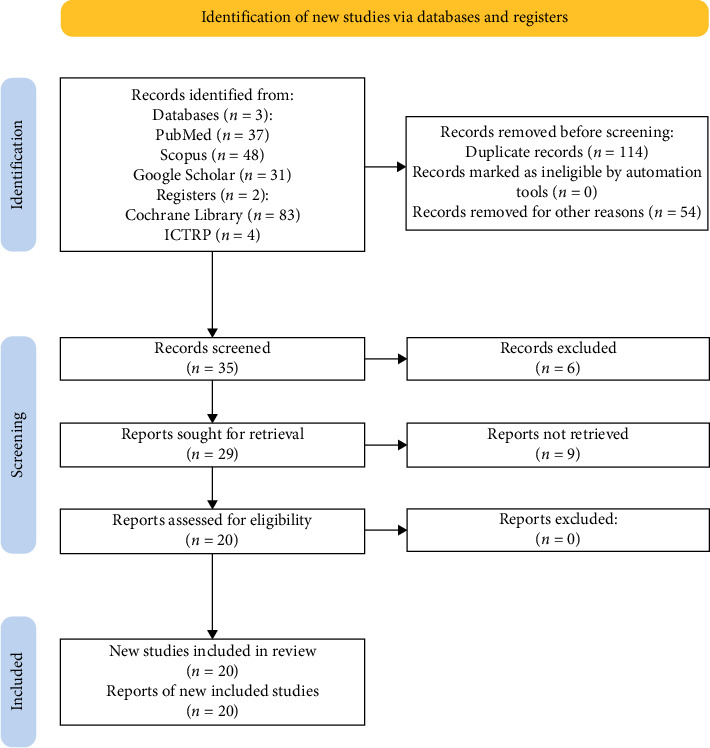
Systematic literature review PRISMA flowchart.

Our search of the databases and registries mentioned earlier yielded a total of 203 studies. After removing 114 duplicates, we screened 89 studies. Fifty-four ineligible studies were removed due to reasons such as irrelevance to the scope of the review or unpublished trials. They included examining the effect of aromatherapy, specifically lavender oil. Mention was made of suppository diclofenac as an analgesic requested by the placebo group for complete analgesia [13]. Similarly, in other studies, suppository diclofenac was an extra analgesic requirement. These studies were more focused on the effects of other agents, such as intrathecal betamethasone, lidocaine, and PCA with morphine and diamorphine [14–19], as well as preassessment tools for pain [20]. Two papers reported severe anaphylactic shock in patients receiving the suppository diclofenac after Cesarean section [21, 22]. One study reported the effect of the suppository diclofenac on shivering instead of pain [23]. One study was ineligible because it considered all other abdominal surgeries instead of caesarean section which is the study population for this systematic review [24]. Of the 35 studies that remained, six studies were ineligible because they were not written in the English language [25–29]. Nine other studies could not be retrieved. Therefore, 20 studies were included in this systematic literature review (Figure 1).

## 3. Results

### 3.1. Descriptive Analysis

**Figure 2:**
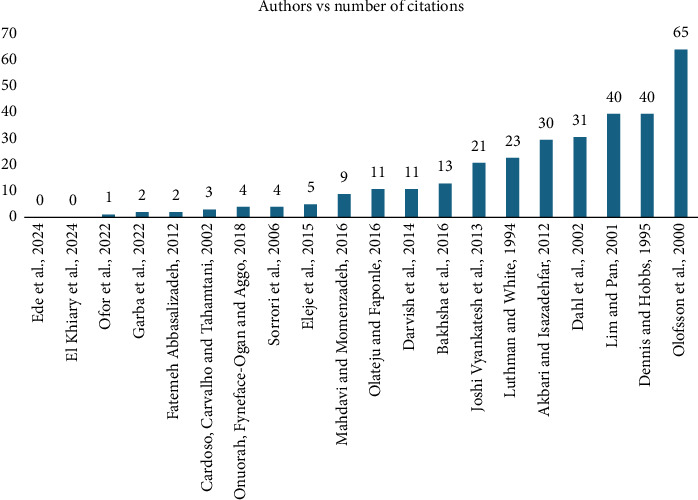
Bar chart of studies against number of citations.

This section provides a descriptive analysis of the articles included in the review.  Figure 2 displays the authors, year of publication, and number of citations. The systematic literature review spans 30 years from 1994 to 2024. The study by Olofsson and colleagues had the most citations [30], followed by that of Lim and colleagues [31] as well as Dennis and colleagues [32].

**Figure 3:**
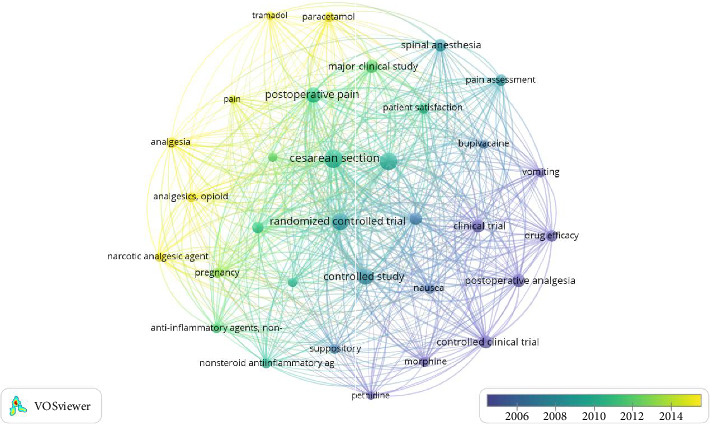
All keywords' networks with VOSviewer.

All the keywords, principally, author and index keywords, are summarized in Figure 3 for co-occurrence. The weight of the frames is based on occurrence. Notable keywords are double-blind procedures, RCT, drug efficacy, and suppository.

**Figure 4:**
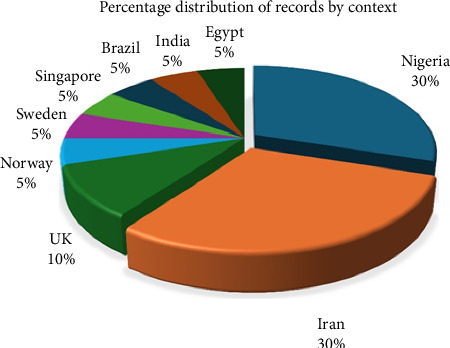
Distribution of the studies by context.

Overview analysis from Biblioshiny indicated that the international coauthorship was 10%, and the average number of citations per document was 15.75. There was no single-authored document. There were 5.6 authors per document. Six studies, representing 30% of the included studies, were conducted in Nigeria and Iran each (Figure 4).

**Figure 5:**
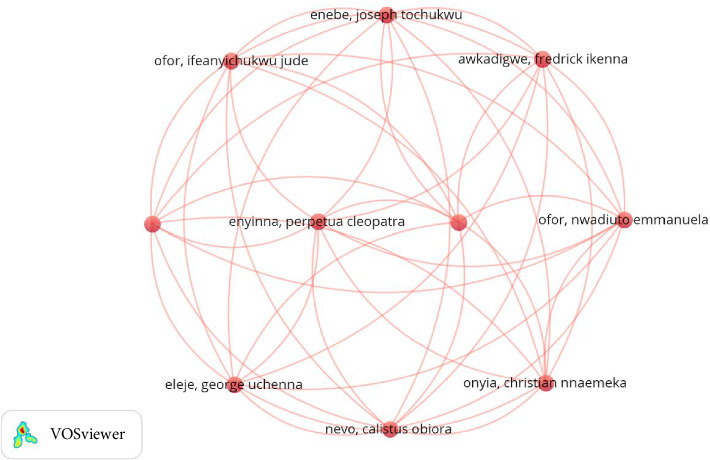
Highest-linked co-authorship network constructed with VOSviewer.

The largest set of connected authors is shown below. It shows a strong community of researchers focused on a common goal (Figure 5).

### 3.2. Literature Classification

#### 3.2.1. Summary Characteristics of the Studies

##### 3.2.1.1. Study Design

Thirteen of the selected studies identified their study design as randomized [30, 31, 33–43] (Table 2). RCTs are the gold standard for studying interventional effects [50, 51]. Assuring the validity and reliability of results in clinical trials requires the fundamental design element of randomization. Randomization minimizes selection bias by randomly allocating individuals to distinct treatment groups, guaranteeing that the groups are comparable at the start of the experiment [52–54]. This approach helps to evenly distribute known and unknown confounding factors among the groups, limiting the potential for these factors to distort the results. The perceived effects of care can be distorted in either way, appearing larger or smaller than they are, if sufficiently disguised random allocation is not used. These distortions can have a size that is equal to or greater than the effects that need to be identified [55].

Consequently, discrepancies in results can be more safely ascribed to the treatment being studied rather than to extraneous factors [51]. In addition to improving the scientific rigor and validity of the trial's conclusions, randomization encourages the use of statistical tests that presuppose random allocation. However, in the case of rare diseases, alternative clinical trials may be undertaken given the difficulty in recruiting participants for such studies [56]. One study reported the use of a quasiexperimental approach [41]. In quasiexperimental studies, participants are not randomly assigned to groups. These studies are used when randomization is not feasible [57], but they are more prone to bias than RCTs, even though some studies state otherwise [58]. Considering the topic of this systematic literature review, Cesarean section did not fall into the category of such rare conditions, as Cesarean section has a high prevalence rate in recent years [59–61]. Therefore, researchers who wish to study the interventional effects of pain relief medicines for Cesarean section should use randomized studies.

Six studies were identified as controlled studies [32, 34–36, 39, 43], while four studies were identified as placebo controlled [32, 34–36] (Table 2). A popular research design in experimental research for ascertaining the impact of a variable or treatment on an outcome of interest is the controlled study [62]. Comparing two or more groups that receive different treatments is what it entails. One group is the experimental group, receiving the intervention or treatment, while the other group is the control group, receiving a placebo or not receiving any treatment at all. In order to minimize bias and establish causal links between variables, this design is crucial [63]. Therefore, controlled studies should include participants who lack exposure or who are without the outcome of interest [64].

Nine studies used the double blinding approach [30–35, 39, 43, 45]. Four studies used a single blinding approach [36–38, 49] (Table 2). In a single-blind study, participants are not familiar with whether they are in the experimental or control group, but the researchers do [65, 66]. Single blinding indicates that the outcomes may be influenced by experimenter effects such as observer bias. However, in a double-blind study, neither the participants nor the researchers know who was in which group. This approach helps prevent bias from both the participants' and the researchers' expectations [65]. Although a meta-epidemiological review of meta-analyses could not find enough evidence of the impact of blinding or nonblinding on effect sizes, it was still recommended that blinding be performed as a methodological safeguard in trials as well as cause-and-effect studies [67].

Two of the selected studies used a comparative study design approach [42, 47] (Table 2). A comparative study is a research approach that focuses on comparing at least two groups, variables or phenomena to determine similarities, differences, and patterns [68]. Such research design is common in a wide range of disciplines, such as sociology, medicine, education, and economics, for examining connections, hypotheses, or the effect of one variable on others. This is essentially an observational study [69]. While controlled studies rely on establishing causality and using manipulation of variables, comparative studies, in the broader sense, tend to concentrate on observation and assessment without changes in the subjects' environment. Comparative studies are very effective; however, they are not without their drawbacks. There is always the issue of confounding factors that are not well addressed, and this would cause bias in the results. However, the use of such data can hinder the elucidation of cause-and-effect relationships because the recorded differences could be due to influences other than those under investigation. Since comparative analysis underpins RCTs, the term “comparative studies” should not be used casually; rather, researchers must be specific about the study design for such interventional studies in Cesarean section [70].

Two studies did not specify the study design by using the terms “randomized”, “double-blind”, “single-blind”, “prospective” or “comparative”; however, the procedures outlined in the patients and methods section alluded to an attempt at a randomized prospective study [44, 46]. Additionally, two studies used the term “clinical trial”, probably due to the unstructured randomization performed in these studies. [48, 49] (Table 2).

##### 3.2.1.2. Interventions

Some hypotheses were revealed in this systematic literature review. Principally, 10 of the studies used diclofenac suppositories independently, while comparing them to either a placebo, another opioid or nonopioid suppository, or intravenous analgesic administration. The hypothesis that diclofenac suppositories reduce extra analgesic requirements was explored in these studies. Three studies compared diclofenac suppository to a placebo [30, 32, 43]. Akbari and Isazadehfar compared it to a placebo in one group and to indomethacin and acetaminophen suppositories in two other groups [45]. Akbari and Isazadehfar specifically highlighted the important role of diclofenac and indomethacin, both of which are NSAIDs, in delaying the time needed before patients request additional pain medicines (opioids) (Table 3).

Two studies explored the concept of the route of administration by using diclofenac suppository and intramuscular (IM) diclofenac [42, 46] (Table 3).

Abbasalizadeh and colleagues compared diclofenac suppository with intramuscular morphine. Intramuscular morphine administration for post-Cesarean section pain is a well-known practice [71]. However, in this study, Abbasalizadeh and colleagues concluded through their findings that diclofenac may be more effective than IM morphine [40]. In contrast, Mahdavi and colleagues used morphine suppositories [49], while Sorrori and colleagues compared with IM pethidine [48].

Eight of the studies considered the concept that the combination of analgesic agents offers better analgesia than independent pain medicine administration [33, 34, 36, 37, 39, 41, 44, 47]. There are differences in the focus on this hypothesis. For instance, Bakhsha and El Khiary assessed the impact of combining diclofenac suppositories with acetaminophen, a widely used analgesic [72], with acetaminophen only. The acetaminophen was administered intravenously, hence, there were different routes of administration but a synergy from the different mechanisms of action of the two agents [33, 47] (Table 3).

Ofor, Eleje, and Ede explored the concept of the adjunctive use of diclofenac suppositories. In their studies, a combination of diclofenac and pentazocine was compared to a combination that received only pentazocine [36, 39, 41]. Garba and colleagues tweaked this concept slightly by comparing the diclofenac-pentazocine combination to the diclofenac-acetaminophen combination [37]. Pentazocine is a mixed-action agonist antagonist of opioid receptors [73]. It is also known to be a mu [74] and kappa receptor agonist [75]. It is more potent than codeine but less potent than morphine [76] and may have fewer hepatotoxic and renal toxic effects [76]. In other conditions, such as acute pancreatitis, pentazocine has been postulated to be a better analgesic than diclofenac [77]. It has been established that this drug possesses a strong degree of analgesia with minimal accompanying psychotomimetic effects or human drug dependency.

One study compared diclofenac suppositories administered independently to another group that received a tramadol suppository [38]. Vegard Dahl also compared a combination of diclofenac and acetaminophen suppositories with another group that received a placebo suppository with an acetaminophen suppository [35]. The query “Do suppositories work the same way; suppository stability and efficacy between opioid-like drugs (tramadol) and nonopioids (diclofenac)” may strike a chord in these studies (Table 3).

Lim and colleagues explored the use of diclofenac as an adjunctive therapy to patient-controlled epidural analgesia (PCEA) [31]. Their findings indicated that the suppository reduces the consumption of ropivacaine and fentanyl, which were the medicinal agents in the PCEA device.

##### 3.2.1.3. Pain Measurements and Rating Scale

Seventeen studies used the VAS as a pain assessment tool, while three [37, 43, 49] used the numerical rating scale (NRS). Vegard Dahl also used an additional verbal rating scale [35]. The VAS is one of the most frequently used tools for assessing changes in the character and intensity of pain [78]. In clinical practice, the percent pain reduction, as evaluated by the VAS, is often used to quantify the effectiveness of treatment [79, 80]. It is also popular in the gynecological space [81, 82] (Table 3).

Both the VAS and NRS are reliable for assessing pain severity, without a significant difference between them [78, 83, 84]. The verbal rating scale is postulated to be less sensitive than the other rating scales [84].

VAS scores can be analyzed using a number of methods, including distribution-based methods, distribution-free methods, categorization methods and descriptive methods only [78]. Aggregated self-reported pain intensity change, as the core index, is employed in most clinical trials and is the ‘gold standard' for measuring change [78]. Another approach to the analysis of VAS scores is to categorize the VAS score into a small number of levels: none, mild, moderate, severe, and unbearable [78]. Nonparametric methods do not specify any probability distribution for the response variable and hence are called distribution-free methods. Rather, they are derived from the orderings or ranks that we assigned to the VAS scores than the actual scores. Hence, they are suitable for ordinal results [78]. When using the VAS, continuous ordinal regression is recommended to have more discriminating power when covariates are highly influential and is thus preferred. Nevertheless, if the spread of the VAS score is not extremely skewed, then more traditional linear methods, such as linear regression (and its analogs), can be used. The Wilcoxon and *t* tests are less powerful by nature than the other tests [78].

##### 3.2.1.4. Findings Reported by the Various Studies

###### 3.2.1.4.1. Diclofenac-Only Pain Management as a Suppository or Injectable Agent

Dennis concluded that diclofenac suppositories prolong the mean time to first analgesia by more than 5 h [32]. Another study concluded that diclofenac suppositories produced better analgesia than morphine suppositories [49]. Moreover, Onuorah reported that suppository diclofenac administered through the rectal route is as efficacious as intramuscular diclofenac injection for post-Cesarean section analgesia, with equal levels of patient satisfaction and acceptability [42]. However, Cardoso expressed a different opinion by demonstrating that when diclofenac is combined with small doses of spinal morphine, its intramuscular administration offers better postoperative analgesia than does the rectal route. Additionally, it was observed in that study that diclofenac seems to have a ceiling effect on the efficacy of this drug when used for immediate postoperative pain management; no advantages were observed with doses larger than 50 mg intramuscularly [46].

###### 3.2.1.4.2. Diclofenac Suppository vs. Other NSAIDs

Khiary concluded that the combination of acetaminophen and diclofenac has stronger and longer analgesic effects than does the single use of each drug [47]. Akbari suggested the use of indomethacin and diclofenac suppositories since there was a significant decrease in pain scores and opioid usage, especially in these groups, compared with the control group [45]. Vegard Dahl concluded that diclofenac suppositories of 100 mg twice daily after Cesarean section are opioid sparing [35]. Bakhsha and Darvish concurred in their conclusion that combination management of pain would have better efficacy in postoperative pain control and cause a reduction in additive analgesia than independent diclofenac suppository management [33, 44].

###### 3.2.1.4.3. Diclofenac Suppository vs. Opioids or Opioid-Like Pain Medicines

Abbasalizadeh found that diclofenac suppositories are significantly more effective than intramuscular morphine suppositories for relieving pain after a Cesarean section [40]. Eleje concluded that the combination of diclofenac and pentazocine significantly alleviated pain, improved patient satisfaction, and promoted earlier mobilization during the post Cesarean period, compared to using pentazocine alone [41]. Ede, Olateju, and Ofor supported this conclusion, emphasizing that adjunctive rectal diclofenac is more effective than pentazocine alone for managing pain after a Cesarean section. They found that fewer patients experienced moderate to severe pain 24–48 h postsurgery, and maternal satisfaction with pain management was higher with diclofenac suppositories [34, 36, 39]. Additionally, while the combination of analgesics offered sufficient pain relief, the pentazocine-diclofenac combination might be linked to extra side effects [37]. Another study indicated that diclofenac suppositories are preferable to tramadol as they do not cause nausea or vomiting and have a longer duration of action [38]. Lim reported that a single 100 mg dose of diclofenac suppository effectively reduces the need for epidural local anesthetics or opioids by 33% during the first 24 h after a Cesarean section [31].

## 4. Discussion

### 4.1. Study Design

This systematic literature review highlighted both strengths and weaknesses worth noting. Regarding the classification of the reviewed literature, most studies employed some form of randomization to eliminate bias; however, omissions were noted in their reporting. Current guidelines for conducting RCTs no longer accept the mere mention of randomization; researchers are expected to provide evidence of its implementation. As such, it is essential for researchers to include key terms related to randomization, such as ‘computer-generated' and ‘concealed allocation to treatment' [55], and to ensure these measures are rigorously applied to minimize the RoB. Additionally, the use of double blinding is strongly recommended.

The term ‘pilot' refers to a specific type of study resembling the intended trial in aspects such as the inclusion of a control group and randomization. In contrast, ‘feasibility' is a broader term used for exploratory studies. Importantly, studies labeled as ‘pilots' must have distinct goals and objectives separate from those of the primary trial, along with a clear plan for further research. It is inappropriate for researchers to label a trial assessing a therapeutic effect as a ‘pilot'.

### 4.2. The Role of Diclofenac in Postoperative Pain Management

Diclofenac suppositories have undoubtedly become a cornerstone of postoperative analgesia. They effectively reduce postoperative pain severity, leading to a prolonged interval before patients request additional analgesics. NSAIDs, including diclofenac, prevent prostaglandin synthesis and have been increasingly used to alleviate pain. By reducing the chemical mediators responsible for painful impulses, these medications indirectly contribute to pain relief, alongside their direct anti-inflammatory and analgesic effects. Moreover, NSAIDs do not cause respiratory depression or other opioid-related side effects, such as ileus, nausea, or drowsiness [48]. Diclofenac's relatively short elimination half-life reduces the risk of drug accumulation within the body [85]. Its advantages as an analgesic include a rapid onset of action and an extended interval to the next required dose. When administered intramuscularly, diclofenac is often as effective as, or more effective than, various narcotic and spasmolytic combinations [46, 85]. Extensive clinical experience has demonstrated its safety profile, confirming that diclofenac is well tolerated compared to other NSAIDs, with a lower risk of stomach ulcers and severe adverse effects. As a result, diclofenac is frequently considered a first-choice NSAID for managing both acute and chronic inflammatory and painful conditions [85–87]. While one review suggested that indomethacin may be superior to diclofenac, the specific route of administration was not clearly stated [88].

Whether diclofenac's effectiveness equals or surpasses that of opioids cannot be confirmed in this systematic review due to insufficient experimental evidence. However, a combination regimen incorporating diclofenac suppositories is strongly recommended, as this approach has been shown to be more effective than using opioids or opioid-like agents alone [25, 89, 90] or any one NSAID alone [91]. Pentazocine and pethidine, for instance, have notable hemodynamic effects, including increases in blood pressure [92]. Opioids are also associated with well-known side effects [90], making reduced opioid use a key objective in most postoperative pain management strategies [93].

### 4.3. Other Findings

The findings of this review showed that combination therapy enhances patient satisfaction and could be an effective substitute for opioids. Based on the type of Cesarean section incurred, it was deduced that elective surgeries accounted for a greater percentage of all surgeries, and a significant proportion of the patients had a history of previous Cesarean section. Some of the limitations of the studies included were the expensive nature of the study and the duration of assessment, which can prolong hospital stays. All the studies were considered as having a high RoB, principally due to the lack of appropriate analysis methods for estimating the effect of assignment to interventions, the absence of randomization techniques and the lack of analysis according to a prespecified plan. However, among these concerns, the issue of randomization techniques, which is the most likely to impact study outcomes, can also be attributed to an omission in the reporting of the research methods section. Since none of the studies significantly deviate from the well-established notion of the benefits of effective multimodal postoperative analgesia, it can be concluded that these omissions in the study design had little to no impact on the study outcomes. Most study findings favor the efficacy of the analgesic combination in the management of pain after Cesarean delivery.

Only three studies stated their study sample as elective Cesarean section patients. The remaining studies used patients who undertook both types of Cesarean section. Incorporating both types of Cesarean section may improve the external validity of results and represent real-world situations. However, combining these categories without making any distinctions may result in inaccurate conclusions on overall safety, effectiveness, or results. Future clinical trials should consider stratifying their results by type of Cesarean section and undertaking subgroup analyses that can help identify specific risks or benefits associated with each type.

### 4.4. Conclusion

The results of this review have several implications for maternal satisfaction and pain management during Cesarean sections. The use of diclofenac suppositories is beneficial. It forms a great component of the multimodal analgesia concept. However, its dosage may have to be standardized to improve therapy.

### 4.5. Limitations of the Review

Some limitations of this review include the reliance on RoB assessments specifically for RCTs, even though not all studies were RCTs. This approach was necessary because most of the included studies were identified as RCTs, and those that did not explicitly state this still exhibited similar characteristics. Furthermore, the review used a limited number of databases and registers, and we were unable to conduct a meta-analysis. The reasons for these decisions are detailed in the methods section.

### 4.6. Strengths of the Review

This systematic literature review highlights important concepts that may have been overlooked by researchers in this field.

### 4.7. Recommendations for Future Research

Long-term data monitoring and patient-oriented methodology can improve the validity of the results and their usability in practice. Long-term outcomes and cost-effectiveness of using diclofenac suppositories and multimodal analgesia can also be explored. In addition, identifying factors that influence treatment outcomes means that future studies will have to apply those aspects in the design of treatment plans to develop more individualized treatments. Studies should distinguish between emergency and elective Cesarean section patients because there could be variations in pain perception depending on the type of Cesarean section and whether it was general anesthesia or spinal anesthesia. There is the need for high-quality randomized trials and investigations into optimal dosing strategies.

## Figures and Tables

**Table 1:** Certainty of assessment.

Certainty assessment							No of patients		Effect		Certainty	Importance
No of studies	Study design	Risk of bias	Inconsistency	Indirectness	Imprecision	Other considerations	Diclofenac suppository	Placebo or other NSAIDs or opioids	Relative (95% CI)	Absolute (95% CI)		
*Pain severity or intensity (assessed with: VAS or NRS; scale from: 0–10)*												
20	Randomized trials	serious^a^	Not serious	Not serious	Not serious	All plausible residual confounding would reduce the demonstrated effect	1221	1098	—	0 (0–0)	⨁⨁⨁⨁ High	IMPORTANT

*Reduced rescue medicine consumption (assessed with: Device or Volume of injectables)*												
13	Randomized trials	serious^a^	Not serious	Not serious	Not serious	All plausible residual confounding would reduce the demonstrated effect	It was expressed as time to request extra pain medicines, or the cumulative dose (mg) of opioids, or opioid-like pain medicines requested by patients				⨁⨁⨁⨁ High	IMPORTANT

*Patient satisfaction (assessed with: NRS or VRS)*												
6	Randomized trials	serious^a^	Not serious	Not serious	Not serious	None	363/1221 (29.7%)	362/1098 (33.0%)	Not estimable		⨁⨁⨁◯ Moderate	IMPORTANT
								0.0%				

*Note:* Question: Diclofenac suppository compared to placebo, other NSAIDs or opioids for postoperative pain after Cesarean section.

Abbreviation: CI, confidence interval.

^a^Most studies were silent on how randomization was performed, lacked the use of preplanned analysis methods and did not estimate the effect of assignment to interventions.

**Table 2:** Literature classification and risk of bias.

Authors	Study design	Context	Focus	Hypothesis	Sample size	Setting	Population	Risk of bias
Bakhsha et al., 2016 [33]	Randomized double-blind clinical trial	Iran	Independent or combination administration of nonopioid analgesics	Combination of analgesic agents offers better analgesia	90	Sayyad shirazi teaching hospital, gorgon, Iran	Cesarean section with spinal anesthesia	High risk of bias
Olateju and Faponle, 2016 [34]	Double-blind randomized placebo-controlled trial	Nigeria	Effective multimodal approach to an existing practice	Combination of analgesic agents offers better analgesia	116	Obafemi Awolowo university teaching hospitals complex, Ile-Ife	Elective or emergency cesarean section with spinal anesthesia	High risk of bias
Darvish et al., 2014 [44]	Not stated	Iran	The efficacy of nonopioids against opioids	Nonopioids(diclofenac and acetaminophen combination) can provide better analgesia to single opioid administration	120	Amiralmomenin hospital, Tehran, Iran	Elective Cesarean section with spinal anesthesia	High risk of bias
Dahl et al., 2002 [35]	Randomized, double-blinded, placebo-controlled study	Norway	NSAIDs (diclofenac suppository) vs opioid (morphine)	Diclofenac suppositories reduce opioid consumption post cesarean section without major effects	82	Baerum hospital	Elective Cesarean section with spinal anesthesia	High risk of bias
Lim and Pan, 2001 [31]	Randomized double-blind study	Singapore	Analgesic efficacy of single dose diclofenac suppository	NSAIDs (diclofenac suppository can reduce PCEA consumption/requirement)	48	Changi General Hospital	Elective Cesarean section with regional anesthesia	High risk of bias
Dennis and Hobbs, 1995 [32]	Double‐blind placebo‐controlled study	UK	NSAIDs (diclofenac suppository) as adjunct postoperative analgesia	NSAIDs (diclofenac suppository) reduces extra analgesic requirements	50	University hospital	Elective Cesarean section with spinal anesthesia	High risk of bias
Akbari and Isazadehfar, 2012 [45]	Double-blind clinical trial study	Iran	Suppositories for postoperative analgesia	Suppositories reduce extra analgesic requirements	120	Ardebil Alavi hospital	Elective Cesarean section with spinal anesthesia	High risk of bias
Ofor et al., 2022 [36]	Single-blind randomized controlled trial	Nigeria	NSAIDs (diclofenac suppository) vs combination of diclofenac suppository and pentazocine	Combination of analgesic agents offers better analgesia	200	ESUT-TH hospital	Elective and emergency Cesarean section with spinal anesthesia	High risk of bias
Garba et al., 2021 [37]	Single-blind, randomized trial.	Nigeria	NSAIDs combinations vs NSAIDs and opioid	Combination of analgesic agents offers better analgesia	193	Usmanu Danfodiyo University Teaching Hospital (UDUTH), Sokoto	Elective or emergency Cesarean section with spinal anesthesia	High risk of bias
Cardoso, Carvalho and Tahamtani, 2002 [46]	Not stated	Brazil	Route and strength of diclofenac for postoperative pain	The route of administration has an impact on the analgesia achieved	186	Hospital Maternidade Santa Joana, são Paulo	Cesarean section with spinal anesthesia	High risk of bias
Joshi Vyankatesh et al., 2013 [38]	Prospective, randomized, single blind	India	Diclofenac suppository vs tramadol suppository	Do suppositories work the same way; suppository stability and efficacy between opioid-like (tramadol) and nonopioids (diclofenac)	60	Hospital	Elective or emergency Cesarean section with spinal anesthesia	High risk of bias
Ede et al., 2024 [39]	Prospective randomized, double blind, placebo-controlled study	Nigeria	NSAIDs (diclofenac suppository) vs. combination of diclofenac suppository and pentazocine	Combination of analgesic agents offers better analgesia	120	Alex Ekwueme Federal University Teaching Hospital, Abakaliki	Elective or emergency Cesarean section with spinal anesthesia	High risk of bias
Fatemeh Abbasalizadeh, 2012 [40]	Randomized clinical trial	Iran	Diclofenac suppository vs morphine IM	NSAIDs (diclofenac suppository) as an alternative to opioids (morphine IM)	120	Taleghani hospital of tabriz	Cesarean section	High risk of bias
Eleje et al., 2015 [41]	Open label, quasirandomized clinical trial	Nigeria	Effective multimodal approach to an existing practice	Combination of analgesic agents offers better analgesia	67	Obstetrics and gynecology complex of the Madonna university, Elele, Nigeria	Cesarean section with general or spinal anesthesia	High risk of bias
El Khiary et al., 2024 [47]	Comparative prospective interventional clinical study	Egypt	Independent or combination administration of nonopioid analgesics	Combination of analgesic agents offers better analgesia	48	Zagazig university Maternity hospital	Cesarean section	High risk of bias
Onuorah, Fyneface-Ogan and Aggo, 2018 [42]	Prospective, randomized, comparative study.	Nigeria	Route and strength of diclofenac for postoperative pain	The route of administration has an impact on the analgesia achieved	88	University of PortHarcourt Teaching Hospital	Cesarean section with spinal anesthesia	High risk of bias
Sorrori et al., 2006 [48]	Clinical trial	Iran	Diclofenac suppository vs pethidine	Alternative analgesia	240	Alzahrah hospital, Isfahan, Iran	Cesarean section patients	High risk of bias
Mahdavi and Momenzadeh, 2016 [49]	Clinical trial	Iran	Comparison between opioid and nonopioid suppository	Suppository stability and efficacy between opioids (morphine) and nonopioids (diclofenac)	100	Shariati hospital, Iran	Elective cesarean section with spinal anesthesia	High risk of bias
Luthman and White, 1994 [43]	Double-blind randomized controlled trial	UK	NSAIDs (diclofenac suppository) as adjunct postoperative analgesia	NSAIDs (diclofenac suppository) reduces extra analgesic requirements	50	Northampton general hospital	Elective Cesarean section patients	High risk of bias
Olofsson et al., 2000 [30]	Randomized double-blind study	Sweden	NSAIDs (diclofenac suppository) as adjunct postoperative analgesia	NSAIDs (diclofenac suppository) reduces extra analgesic requirements	50	Karolinska hospital, SE-17176, Stockholm, Sweden	Elective Cesarean section with spinal anesthesia	High risk of bias

**Table 3:** Interventions and pain measurement characteristics.

Authors	Tools	Number of pain measurements	Duration of pain measurements (h)	Diclofenac only	Combination drug	Comparison drugs-1	Comparison drugs-2	Strength of diclofenac suppository	Frequency of administration of diclofenac suppository
Bakhsha et al., 2016 [33]	VAS	6	24	Yes	Yes with 500 mg IV acetaminophen	1000 mg IV acetaminophen	None	100 mg	Not stated
Olateju and Faponle, 2016 [34]	VAS	4	24	No	Yes with 30 mg IM pentazocine 6 hourly	30 mg IM pentazocine 6 hourly alone	None	100 mg	12 hourly
Darvish et al., 2014 [44]	VAS	Not stated	Not stated	No	Yes with 1000 mg IV acetaminophen	20 mg IM meperidine (pethidine)	None	Not stated	4 hourly
Dahl et al., 2002 [35]	VAS & VRS	9	32	No	Yes with 1000 mg acetaminophen suppository	Placebo with 1000 mg acetaminophen suppository	None	100 mg	12 hourly
Lim and Pan, 2001 [31]	VAS	4	24	No	Yes, with PCEA (ropivacaine and fentanyl)	No suppository with PCEA (ropivacaine and fentanyl)	None	100 mg	Single administration
Dennis and Hobbs, 1995 [32]	VAS	8	48	Yes	No	Placebo	None	100 mg	Single administration then after 16 h
Akbari and Isazadehfar, 2012 [45]	VAS	3	24	Yes	No	Placebo	Acetaminophen (325 mg) and indomethacin (50 mg) suppositories	50 mg	6 hourly
Ofor et al., 2022 [36]	VAS	5	48	No	Yes with 30 mg IM pentazocine 6 hourly	60 mg IM pentazocine 6 hourly	None	100 mg	12 hourly
Garba et al., 2021 [37]	NRS	4	24	No	Yes with 60 mg IM pentazocine	600 mg IM acetaminophen 6 hourly and 100 mg diclofenac suppository	None	100 mg	12 hourly
Cardoso, Carvalho and Tahamtani, 2002 [46]	VAS	12	6	Yes	No	50 mg IM diclofenac	75 mg IM diclofenac	50 mg	Single administration
Joshi Vyankatesh et al., 2013 [38]	VAS	10	10	Yes	No	100 mg tramadol suppository	None	100 mg	Single administration
Ede et al., 2024 [39]	VAS	4	48	No	Yes with 30 mg IM pentazocine 6 hourly	Anusol suppository plus 30 mg IM pentazocine	None	75 mg	12 hourly
Fatemeh Abbasalizadeh, 2012 [40]	VAS	3	24	Yes	No	10 mg IM morphine	None	100 mg	8 hourly
Eleje et al., 2015 [41]	VAS	Not stated	Not stated	No	Yes with 30 mg IM pentazocine	30 mg IM pentazocine 6 hourly alone	None	100 mg	12 hourly
El Khiary et al., 2024 [47]	VAS	6	24	Yes	Yes with 500 mg IV acetaminophen	1000 mg IV acetaminophen	None	100 mg	Single administration
Onuorah, Fyneface-Ogan and Aggo, 2018 [42]	VAS	11	24	Yes	No	100 mg IM diclofenac	None	100 mg	Single administration
Sorrori et al., 2006 [48]	VAS	4	26	Yes	No	IM pethidine	None	100 mg	8 hourly
Mahdavi and Momenzadeh, 2016 [49]	NRS	9	24	Yes	No	Morphine suppository 10 mg	None	100 mg	Single administration
Luthman and White, 1994 [43]	Quasi NRS	10	24	Yes	No	Placebo	None	100 mg	Single administration
Olofsson et al., 2000 [30]	VAS	12	12	Yes	No	Placebo	None	50 mg	8 hourly

Abbreviations: VAS, visual analog scale; NRS, numerical rating scale.

## Data Availability

Data related to the review have been deposited for review into Current Research in Health Sciences database under the Zenodo repository and are available at following URL: https://doi.org/10.5281/zenodo.14732125.

## Supporting Information

Supporting Information 1. Pain Scores of patients in Diclofenac suppository-only groups

Supporting Information 2. Key findings and Discrepancies identified in the selected research articles
