# Supplementary material for: The Impact of Diclofenac Suppositories on Post-Cesarean Section Pain: A Systematic Literature Review
Source: Anesthesiol Res Pract. 2025 Mar 16;2025:5457722. doi: 10.1155/anrp/5457722 (PMC11930387; doi:10.1155/anrp/5457722)
Supplement: Supporting Information — Additional supporting information can be found online in the Supporting Information section. [file 5457722.f1.docx]

Supplementary 1. Pain Scores of patients in Diclofenac suppository-only groups

| **Authors** | **Pain scores** | | | | | | | | | | |  |
| --- | --- | --- | --- | --- | --- | --- | --- | --- | --- | --- | --- | --- |
|  | **0** | **1** | **2** | **4** | **6** | **8** | **10** | **12** | **16** | **18** | **24** | **26** |
| Bakhsha *et al.*, 2016 |  |  | 4.33±1.6 | 4.53±1.27 | 5.13±1.4 |  |  | 4.43±1.17 |  |  | 3.03±1.02 |  |
| Akbari and Isazadehfar, 2012 |  | 4.73±2.16 |  |  |  |  |  | 1.97±1.38 |  |  | 0.97±0.85 |  |
| Dennis and Hobbs, 1995 | 3.93 | 9 | 8.88 | 6.42 | 9.13 |  |  | 5.93 |  |  | 7.4 |  |
| Cardoso, Carvalho and Tahamtani, 2002 |  | 1.35±1.43 | 1.28±1.22 | 0.50±0.78 | 0.36±0.82 |  |  |  |  |  |  |  |
| Joshi Vyankatesh *et al.*, 2013 |  |  | 0.6 ±0.5 | 2.10±0.55 | 2.63±1.02 | 2.07±0.98 | 2.57±0.73 |  |  |  |  |  |
| Fatemeh Abbasalizadeh, 2012 |  |  |  |  |  | 5.1±0.90 |  |  | 3.02±1.17 | | 1.38±0.90 |  |
| El Khiary *et al.*, 2024 |  |  | 3.8±0.8 | 4.8±0.8 | 5.3±0.7 |  |  | 4.2±0.6 |  |  | 3.1±0.4 |  |
| Onuorah, Fyneface-Ogan and Aggo, 2018 |  |  |  |  |  |  |  |  |  |  |  |  |
| Sorrori *et al.*, 2006 |  |  | 6.8±2.3 |  |  |  | 2.05±2.07 |  |  | 1.4±1.6 |  | 0.5±1.1 |
| Mahdavi and Momenzadeh, 2016 |  |  |  |  |  |  |  | 3.63±0.96 |  |  | 3.63±0.96 |  |
| Luthman and White, 1994 |  |  |  |  |  |  |  |  |  |  |  |  |
| Olofsson *et al.*, 2000 |  |  |  |  |  |  |  |  |  |  |  |  |

*Measured using the VAS

Supplementary 2. Key findings and Discrepancies identified in the selected research articles

| **Authors** | **Primary outcome** | **Secondary outcome** | **Key Findings** | **Discrepancies** |
| --- | --- | --- | --- | --- |
| Bakhsha *et al.*, 2016 | Pain severity | Duration of analgesia, request for more pain medicines | Combination management of pain is better than independent diclofenac suppository management | Frequency of administration of intervention is not stated but appears to be a one-time, whether it was pre-op or post-op is also not stated. Concerns about comparing different routes of administration. |
| Olateju and Faponle, 2016 | Pain at rest and on movement | side effects, request for rescue pain medicines | The addition of diclofenac suppository to intramuscular pentazocine provides better pain relief after cesarean section and increased patient satisfaction. |  |
| Darvish *et al.*, 2014) | Pain | side effects, request for rescue pain medicines | Paracetamol and Diclofenac combination would have a better efficacy in postoperative pain control and need reduction to additive analgesia compared to Meperidine. | Strength of diclofenac suppository used was not stated, side effects wrongly stated as adverse effects |
| Dahl *et al.*, 2002 | Pain at rest | Side effects and request for rescue pain medicines (IV Morphine) | Diclofenac group used less morphine postoperatively, showing opioid-sparing effect. No significant differences in demographic data, side effects, or discharge time. Diclofenac suppositories 100 mg twice daily after caesarean section are opioid sparing. | Small sample size affected statistical significance of results. Inability to use repeated measures of ANOVA due to unequal variance |
| Lim and Pan, 2001 | PCEA consumption | Patient satisfaction and pain scores on movement | A single administration of 100 mg diclofenac suppository is effective in reducing post-Cesarean epidural local anesthetic/opioid requirements by 33% for the first 24 hr post-operatively. | Small sample size |
| Dennis and Hobbs, 1995 | Pain at rest and on movement | side effects and request for rescue medicines (IM morphine and oral co-proxamol) | Diclofenac prolonged the mean time to first analgesia by more than 5h from 13 h 45min in the placebo group to 18h 58min @ < 0.03) | VAS score may not be so significantly different from each other. Small sample size |

Supplementary 2. *Continued*

| **Authors** | **Primary outcome** | **Secondary outcome** | **Key Findings** | **Discrepancies** |
| --- | --- | --- | --- | --- |
| Akbari and Isazadehfar, 2012 | Pain severity | Opioid consumption | There is significant decreased pain scores and opioid usage especially in indomethacin and diclofenac groups rather than control group. |  |
| Ofor *et al.*, 2022 | Pain perception | Patient satisfaction and request for rescue pain medicines | Rectal diclofenac combined with intramuscular pentazocine was significantly better at controlling pain compared with pentazocine alone in the first 48 h following caesarean section. |  |
| Garba *et al.*, 2021 | Pain severity | Side effects and request for rescue medicines (IM Piroxicam) | Both combination of analgesics provided adequate analgesia but pentazocine + diclofenac combination had better pain relief but was more associated with side effects. |  |
| Cardoso, Carvalho and Tahamtani, 2002 | Pain severity | Request for rescue pain medicines (30mg IV meperidine) | The results of this study demonstrate that while combined with small doses of spinal morphine, the intramuscularly administration of diclofenac offers better postoperative analgesia than the rectal route. Additionally, it seems that there is a ceiling effect for this drug, used for immediate postoperative pain management; no advantages are observed with doses larger than 50 mg intramuscularly. | Contrary findings when compared to the remaining studies. |
| Joshi Vyankatesh *et al.*, 2013 | Pain | Side effects and request for rescue pain medicines (IV Pentazocine) | Rectal suppository of tramadol as well as diclofenac are effective for postoperative analgesia in Cesarean section. Diclofenac is better alternative than tramadol as it is devoid of nausea and vomiting and have longer duration. |  |

Supplementary 2. *Continued*

| **Authors** | **Primary outcome** | **Secondary outcome** | **Key Findings** | **Discrepancies** |
| --- | --- | --- | --- | --- |
| Ede *et al.*, 2024 | Pain at rest | Time from surgery to ambulation, Passage of flatus, maternal satisfaction and presence of complications. | Adjuvant rectal diclofenac is superior to pentazocine alone in the management of pain after caesarean section. Less number of patients had moderate to severe pain at 24 hours post operation. Maternal satisfaction in relation to pain management is better with diclofenac suppository |  |
| Fatemeh Abbasalizadeh, 2012) | Pain | None | The current study showed that the diclofenac suppository is considerably more efficient than IM morphine in relieving post-CS pain. |  |
| Eleje *et al.*, 2015 | Pain | Satisfaction with pain relief | Diclofenac combined with pentazocine significantly reduced pain, increased patients’ satisfaction and earlier mobilization during post-Cesarean period compared to pentazocine alone. |  |
| El Khiary *et al.*, 2024 | Pain | None | The combination of acetaminophen and diclofenac has stronger and longer analgesic effects than the single use of each drug | Single administration of interventions |
| Onuorah, Fyneface-Ogan and Aggo, 2018 | Pain | Satisfaction with pain relief and request for rescue pain medicine (IM Pentazocine) | Suppository diclofenac administered through the rectal route is as efficacious as intramuscular diclofenac injection for post Caesarean section analgesia with equal levels of patient satisfaction and acceptability. | Single administration of interventions |
| Sorrori *et al.*, 2006 | Pain | Side effects, Satisfaction with pain relief | results of the present study showed that the use of suppository diclofenac is an appropriate replacement therapy for pain relief after C/S. | The dosage frequency is higher than normal. Side effects were not mentioned with such high frequency dosing |

Supplementary 2. *Continued*

| **Authors** | **Primary outcome** | **Secondary outcome** | **Key Findings** | **Discrepancies** |
| --- | --- | --- | --- | --- |
| Mahdavi and Momenzadeh, 2016 | Pain Severity | Request for more pain medicines (IM pethidine) | Diclofenac suppository provides better pain relief compared to morphine suppository in the first 12hrs postoperative with delayed request for more pain medicines | Single administration of interventions |
| Luthman and White, 1994 | Morphine request and consumption | Pain | Morphine consumption reduced but pain score remains same between placebo group and diclofenac suppository group | Research method not stated, dose of suppository diclofenac not stated, how postoperative pain was measured is not stated either |
| Olofsson *et al.*, 2000 | Pain intensity | Request for more pain medicines (IM Ketobemidone via PCA) and patient satisfaction | A multimodal analgetic strategy with the addition of 150 mg diclofenac during the first 24 h after CS reduces the need for opioids significantly with maintained or improved analgetic effect. This is expected to reduce the risk of negative side-effects of systemic opioids. | Limited time for pain measurements |
